# Clinical Correlates of In-Hospital Mortality in Patients Undergoing Inferior Vena Cava Filter Placement for Acute Deep Vein Thrombosis

**DOI:** 10.3390/jcm13082285

**Published:** 2024-04-15

**Authors:** Enrica Porceddu, Rosa Talerico, Gabriele Ciasca, Giulia Cammà, Riccardo Di Santo, Matilde Peri, Alessandro Cina, Roberto Pola, Angelo Porfidia

**Affiliations:** 1 Thrombosis Unit, Department of Geriatric, Orthopedic, and Rheumatologic Sciences, Fondazione Policlinico Universitario A. Gemelli IRCCS, Università Cattolica del Sacro Cuore, 00168 Rome, Italy; 2 Department of Neuroscience, Fondazione Policlinico Universitario A. Gemelli IRCCS, Università Cattolica del Sacro Cuore, 00168 Roma, Italy; 3 Department of Radiology, Fondazione Policlinico Universitario A. Gemelli IRCCS, Università Cattolica del Sacro Cuore, 00168 Roma, Italy

**Keywords:** IVCF placement, acute DVT, in-hospital mortality

## Abstract

**Background:** It is reasonable to place an Inferior Vena Cava Filter (IVCF) when an acute deep vein thrombosis (DVT) of the lower limbs occurs in a patient with absolute contraindication to therapeutic anticoagulation. An additional potential reason for placing an IVCF is the need to stop therapeutic anticoagulation in a patient with acute DVT who must undergo urgent non-deferrable surgery. However, IVCFs are often used outside of such established indications and many authors argue about their actual utility, especially in terms of survival. In this retrospective study, we looked for clinical correlates of in-hospital mortality among patients who underwent IVCF placement, limiting our analysis to the cases for which a correct indication to IVCF placement existed. **Methods**: We retrospectively analyzed the electronic database of our University Hospital, searching for consecutive hospitalized patients who had acute DVT and underwent IVCF placement because of an established contraindication to therapeutic anticoagulation and/or because it was necessary to stop anticoagulation due to urgent surgery. The search covered the period between 1 January 2010 and 31 December 2020. **Results:** The search resulted in the identification of 168 individuals. An established contraindication to therapeutic anticoagulation was present in 116 patients (69.0%), while urgent non-deferrable surgery was the reason for IVCF placement in 52 patients (31.0%). A total of 24 patients (14.3%) died during the same hospital stay in which the IVCF was placed. Mortality rate was significantly higher in patients with a contraindication to anticoagulation than in patients who underwent IVCF placement because of urgent surgery (19.0% vs. 3.8%, OD 5.85 vs. 0.17). In-hospital mortality was also significantly higher among patients with chronic kidney disease and those who needed blood cell transfusion during hospitalization. **Conclusions:** This study provides novel information on clinical correlates of in-hospital mortality among patients with acute DVT who undergo IVCF. Prospective observational studies are needed to substantiate these findings.

## 1. Introduction

The standard treatment for deep vein thrombosis (DVT) of the lower limbs is anticoagulation at therapeutic doses [1,2]. However, management becomes more complicated when DVT occurs in patients with strong contraindications to anticoagulation [3]. An example is patients with active or recent major bleeding, low platelet count, or severe coagulopathies. In these situations, it might be necessary to withhold therapeutic anticoagulation. A strategy that is sometimes used in these patients is to place a filter in the inferior vena cava, to prevent pulmonary embolism (PE) [4,5,6,7]. A similar strategy is sometimes used in patients with a DVT of the legs who must undergo urgent surgery and, therefore, need to temporarily stop therapeutic anticoagulation [3,8,9]. Nonetheless, in the real world, inferior vena cava filters (IVCFs) are often used inappropriately [10,11]. In addition, although most IVCFs are nowadays retrievable, their removal rate is low, with increased risk of morbidity in patients with filters that remain in place [12]. Finally, evidence of clear benefits of IVCF placement on important clinical outcomes, such as recurrent PE and death, is lacking. Indeed, many authors have reported no significant differences in survival in patients with versus without placement of an IVCF [13,14]. In general, mortality in these patients appears to be high, ranging from 7 to 14% [5,6,15], but most studies have included patients who did not have an established indication to IVCF placement, and sometimes did not even have an acute venous thrombosis [16]. In this scenario, it is difficult to establish whether the use of IVCFs is advantageous or futile, or even deleterious, in some patients, even in the short term.

The aim of this study was to look for clinical correlates of in-hospital mortality among patients who underwent IVCF placement, limiting our analysis to individuals who either had an established and well-documented contraindication to full-dose anticoagulation and/or required temporary suspension of therapeutic anticoagulation because of urgent surgery that could not be postponed.

## 2. Materials and Methods

We carried out a retrospective analysis of the electronic database of the “Fondazione Policlinico Universitario Agostino Gemelli IRCCS”, looking for patients hospitalized between 1 January 2010 and 31 December 2020 who met the following criteria: (i) had an established diagnosis of acute DVT of the lower limbs; (ii) underwent placement of an IVCF within 4 weeks from DVT diagnosis; (iii) had an established and well-documented contraindication to full-dose anticoagulation and/or required temporary suspension of therapeutic anticoagulation because of urgent surgery that could not be postponed.

The diagnosis of DVT of the lower limbs had to be performed by vascular ultrasound at a precise date. The 4-week time frame that we chose between the diagnosis of DVT and the placement of the IVCF accounts for the fact that full-dose anticoagulation is mandatory during at least the first 4 weeks of DVT therapy [3]. Therefore, we decided that the need to withhold therapeutic anticoagulation during this time frame might constitute a reasonable indication to IVCF placement. The following were considered valid criteria to determine that a patient had contraindication to anticoagulation:

- recent (within 14 days from the diagnosis of DVT) or active major bleeding (MB) or clinically relevant non-major bleeding (CRNMB), as defined by the International Society of Thrombosis and Haemostasis (ISTH) [17,18];
- platelet count < 25 × 10^9^/L; (normal range 150–450 × 10^9^/L);
- presence of diseases/lesions associated with “high bleeding risk”. The definition of “high bleeding risk” is extremely heterogeneous in the literature without a unanimous consensus. In our study, we considered at “high bleeding risk” patients with one or more of the following pathological conditions: MB and/or CRNMB that occurred between 15 and 30 days before the diagnosis of DVT and did not receive a definitive treatment of the underlying cause; any hematological and/or coagulation disorder associated with increased bleeding risk; primary or secondary tumors of the brain; advanced liver cirrhosis with portal hypertension and esophageal varices; stroke or transient ischemic attack in the previous 6 months [19,20].

The following demographic and clinical characteristics were analyzed for each patient: age, sex, comorbidities, assumption of chronic antithrombotic therapy (either antiplatelets and anticoagulants), laboratory parameters before IVCF placement (hemoglobin levels, platelet count, creatinine), indication to IVCF placement, type of IVCF, and, if it was the case, resumption of anticoagulant treatment after IVCF placement. Regarding chronic kidney disease (CKD), it was defined as creatinine clearance <50 mL/min (normal value ≥ 90 mL/min/1.73 m^2^). Regarding the indication to IVCF placement, a distinction was made between patients who had contraindication to anticoagulation and those who needed to temporarily stop anticoagulation because of urgent surgery. A comparison was made between patients who died and those who did not die during hospitalization. A multivariate analysis with a logistic regression was then performed to identify factors associated with mortality.

### Statistical Analysis

The distribution of data for the continuous variables considered in the study was assessed with the aid of the Shapiro–Wilk test and by means of a qualitative analysis of the QQ plots. This analysis showed the presence of significant deviations from normality in several cases. Therefore, continuous variables are discussed in terms of median and interquartile range. Categorical variables are presented in the form of frequency expressed as a percentage. The differences between the continuous variables of the patient groups were assessed using the non-parametric Wilcoxon–Mann test for independent samples, and those for the categorical variables by means of the 2’s or Fisher’s test. Statistical significance was achieved when the *p*-value was less than 0.05.

The data were analyzed univariately and multivariately. In the first case, the odd ratios (OR) and the corresponding 95% confidence intervals were calculated using the equation OR = ad/bc ± 1.96√(1/a + 1/b + 1/c + 1/d). An OR value consistent with 1 indicates the absence of a significant association between the variables considered. In the second case, the OR values were calculated from the coefficients obtained by means of a multivariate logistic regression. The subset of clinical, laboratory, and demographic variables most significant for classifying in patients belonging to the two groups under study were selected by backward/forward stepwise regression using Aikake’s information criterion minimization AIC = 2k − 2 ln (L), where L is the maximum working likelihood and k represents the number of parameters included in the model. The statistical analysis was conducted using version 4.0.2 (22 June 2020) of the open-source software R. and version 15.32 of Microsoft Excel for Mac 2017. The study was conducted following the STROBE guidelines for observational studies and was carried out as part of protocol number 49904/18, approved by the Ethics Committee of the Fondazione Policlinico Universitario A. Gemelli IRCCS.

## 3. Results

### 3.1. General Characteristics

Our search led to the identification of 168 patients who met the criteria to be included in the analysis. Their clinical and demographic characteristics are presented in Table 1.

The median age was 67 years. There were 72 males (42.8%) and 96 females (57.2%). The following comorbidities were present: history of VTE in 19 patients (11.3%); atrial fibrillation in 11 patients (6.5%); arterial hypertension in 68 patients (40.5%); type 2 diabetes mellitus in 27 patients (16.1%); CKD in 16 patients (9.5%); history of coronary artery disease (CAD) in 8 patients (4.8%); history of cerebrovascular disease (CVD) in 8 patients (4.8%); chronic anemia in 10 patients (5.9%); previous bleeding in 16 patients (9.5%); cancer, active or previous, in 99 patients (58.9%); hematological disease in 11 patients (6.5%).

Regarding the site of DVT, 144 patients (85.7%) had a proximal DVT. The remaining 24 patients (14.3%) had multiple distal vein thrombosis of the legs.

Regarding the indication to IVCF placement, 116 patients (69.0%) had a contraindication to anticoagulation, while 52 patients (31.0%) underwent IVCF placement because they had to temporarily stop anticoagulation due to urgent surgery that could not be postponed. Of note, urgent surgery was needed in a total of 101 patients (60.1%) in our population, but in many of them (*n* = 49) the indication to IVCF placement was due to a pre-existing contraindication to anticoagulation and not surgery itself. Reasons for which anticoagulation was contraindicated in our cohort were: recent or active MB or CRNMB (*n* = 82, 48.8%), severe thrombocytopenia (*n* = 20, 11.9%), and presence of diseases/lesions at high hemorrhagic risk (*n* = 46, 27.4%). Among the latter, 13 patients had a previous bleeding from the localization of cancer (bladder, colon, uterus, lung) without definitive treatment of the underlining cause; 13 patients had a primary or secondary cerebral cancer; 3 patients had a history of gastrointestinal recurrent bleeding due to angiodysplasia or ulcerative colitis or gastroesophageal varices; 6 patients were hospitalized for or had a recent history of ischemic or hemorrhagic stroke.

Regarding the 82 recent or active bleedings, 70 (85.3%) were MBs and 12 (14.7%) were CRNMBs. Sites of bleeding were the gastrointestinal tract (*n* = 24, 14.3%), the central nervous system (brain) (*n* = 25, 14.9%), the genital system (uterus) (*n* = 5, 3.0%), and the urinary system (*n* = 3, 1.8%). Fifty-eight patients (34.5%) had received blood transfusions.

The median hemoglobin value was 10.5 g/dL, median platelet count was 245 × 10^9^/L, median plasma creatinine level was 0.8 mg/dL, and median fibrinogenemia levels were 404 mg/dL.

Regarding therapy, 32 patients (19.0%) were on chronic antithrombotic therapy. Specifically, 15 patients were on antiplatelet therapy (8.9%) and 17 were on anticoagulant therapy (10.1%). In the days that followed IVCF placement, 47 patients (28.0%) started (or resumed) anticoagulant therapy at full dose. A smaller number of patients started (or resumed) anticoagulation at an under-therapeutic dose (*n* = 39, 23.2%) or at a prophylactic dose (*n* = 24, 14.3%). Fifty-eight patients (34.5%) did not receive any anticoagulant therapy after IVCF placement.

Regarding the type of filters, 71 were definitive (42.2%) and 97 (57.8%) were retrievable. Of these, only 12 were removed during hospitalization because the indication to filter maintenance had ceased.

### 3.2. In-Hospital Mortality

The 168 patients in our cohort were divided into two groups: a first group consisted of 24 patients (14.3%) who died during hospitalization and a second group consisted of 144 patients (85.7%) who did not die during hospitalization.

Of the 24 patients who died during the same hospital stay in which the IVCF was placed, 17 (70.8%) died within 30 days after IVCF placement. Of these, 10 died from sepsis, 2 from terminal neoplasia, 3 from the consequences of hemorrhages (one spleen rupture and two major gastrointestinal bleedings), and 2 from the consequences of massive pulmonary embolism (PE) already present before the placement of the vena cava filter. Of the 7 patients who died between 30 and 60 days, 5 died from sepsis, 1 from terminal neoplasia, and 1 from the consequences of a ruptured brain aneurysm.

In Table 2, we present the results of a comparative analysis of continuous variables between patients who died and those who did not die during hospitalization.

The presence of anemia prior to IVCF placement was significantly more frequent among patients who died during hospitalization (*p* = 0.008). There were instead no differences between the two groups in terms of age, platelet count, creatinine, and fibrinogen values.

In Table 3 we present the results of a univariate analysis, conducted on all binary clinical and demographic variables previously reported in Table 1.

Chronic kidney disease (OR, 4.47; 95%CI, 1.45–13.76; *p* = 0.009), the presence of any contraindication to anticoagulation (OR, 5.85; 95%CI, 1.32–25.9; *p* = 0.02), recent bleeding (OR, 2.95; 95%CI, 1.15–7.55; *p* = 0.02), particularly gastrointestinal bleeding (OR, 3.08; 95%CI, 1.11–8.49; *p* = 0.03), and the need for blood transfusion (OR, 3.91; 95% CI, 1.59–9.63; *p* = 0.003) were found to be associated with in-hospital mortality. On the other hand, recent surgery was negatively associated with in-hospital mortality (OR 0.34; 95% CI, 0.14–0.83; *p* = 0.01).

**Figure 1:**
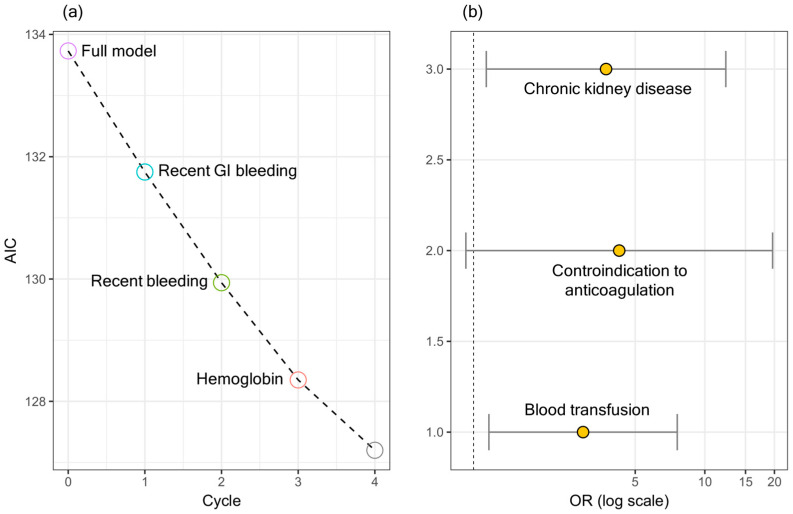
Results of a multivariate analysis on significant variables in Table 2 and Table 3. Stepwise backward/forward regression to select variables (**a**). The ORs and the corresponding confidence intervals for the selected variables (**b**).

Figure 1a shows a stepwise backward/forward regression, which led to the selection of the following variables at the end of the minimization runs: CKD, blood transfusion, and contraindication to anticoagulation. Figure 1b shows the ORs and the corresponding confidence intervals for the selected variables.

The ORs and the corresponding confidence intervals are summarized in Table 4. A significant association can be seen between in-hospital mortality and CKD and blood transfusion.

## 4. Discussion

The goal of placing an IVCF is to trap fragmented emboli travelling from the deep veins of the legs to the pulmonary circulation. This could be of theoretical benefit in patients with acute DVT who cannot be given anticoagulation because of high risk of bleeding. The problem is that IVCFs are often used inappropriately. For instance, they have been (and in some centers still are) used prophylactically, in patients with no DVT who undergo major spine surgery, or in trauma patients, although there is substantial evidence to indicate that this practice is useless, with no statistical difference in PE or mortality rates, and even potentially deleterious to patients, with increased incidence of DVT [16]. A recent systematic review and meta-analysis has shown a significantly decreased utilization of IVCF in hospitals equipped with pulmonary embolism response teams (PERTs) [21], compared to hospitals without PERTs, demonstrating that expertise goes along with appropriate use of filters. Nonetheless, even when IVCFs are properly used, their clinical efficacy profile is still unclear. A recently updated Cochrane review found only six studies that could be examined regarding the efficacy of IVCFs [22]. Of these, only two were both applicable in current clinical settings and of good methodological quality. One was a randomized open-label trial studying the effect of a retrievable IVCF plus anticoagulation versus anticoagulation alone on risk of recurrent PE in 399 participants over 3 months. There was no evidence of a difference in the rates of PE, death, lower extremity DVT, or bleeding at three and six months after the intervention. The second clinically relevant study was a randomized open-label trial of 240 participants who had sustained multiple traumatic injuries, allocated to a filter or no filter three days after injury, in conjunction with anticoagulation and intermittent pneumatic compression. Prophylactic anticoagulation was initiated in both groups when it was thought safe. There was no evidence of a difference in symptomatic PE, death, or lower limb venous thrombosis rates. The remaining four studies were heterogeneous. This review indicates that no firm conclusions can be drawn on the efficacy of IVCFs and their potential impact on survival. More recently, survival outcomes after IVCF placement have been assessed in cancer patients [23], with the demonstration that the median survival following filter placement was only 2 (1.45–2.55) months for patients with advanced-stage solid tumors, and 5 (0.62–9.38) months for patients with brain tumors. These findings further support the concept that IVC filter placement offers limited benefits to patients with advanced-stage disease and therefore the decision to place an IVCF should be carefully weighted.

In this scenario, our study has several strengths. First, it includes only subjects with well-documented indications to IVCF placement, i.e., presence of acute DVT of the legs and concomitant contraindication to anticoagulation (for a specified reason) and/or need to temporarily stop anticoagulation to undergo urgent surgery. This makes our population, although relatively small, very homogeneous, and particularly valuable, compared to what is present in the literature. Second, it provides updated information on the clinical and demographic characteristics of patients requiring IVCF placement. Our results show that about half of these patients have active or previous cancer and about 10% have a previous history of VTE. In half of the cases there is a recent bleeding and approximately one-third have received at least one blood cell transfusion. Our results also show that only a small portion of patients resume (or start) anticoagulation at full dose after IVCF placement, and that this is extremely rare among subjects who underwent filter placement because of a contraindication to anticoagulation. Finally, our study identifies clinical correlates of in-hospital mortality. This was achieved by comparing clinical, laboratory, and therapeutic characteristics of patients who died with those of the patients who did not die during hospitalization. The presence of CKD and the need for blood cell transfusion were found to be factors associated with an increased risk of in-hospital mortality. Contraindication to anticoagulation, after the minimization process of the analysis, although showing an OR of about four, was not statistically associated with in-hospital mortality. However, further studies with larger sample sizes are needed to ensure that this parameter is not an additional clinical correlate of in-hospital mortality in this category of patients. Indeed, the group with a contraindication to anticoagulation had a mortality rate of 19.0%, which was significantly higher than the 3.8% that was registered in the population who underwent IVCF placement because of a temporary suspension of anticoagulation due to urgent surgery.

Our study has some limitations. The number of patients examined is limited and, therefore, our results need to be confirmed in numerically larger samples. Furthermore, it has the limitations inherent to the retrospective nature of the study. Nonetheless, this is, to our knowledge, the first study that tries to identify factors associated with in-hospital mortality in the peculiar population of subjects who undergo placement of an IVCF for acute VTE.

## 5. Conclusions

IVCFs are potentially useful devices to prevent PE in patients with DVT who cannot be appropriately treated with anticoagulation. However, they are often inappropriately used, and it is not clear whether they have a beneficial impact on clinical outcomes, and in which patients. This study identifies CKD and need for blood cell transfusion as predictors of in-hospital mortality among patients with acute VTE who undergo IVCF placement and has the strength of having included only subjects with a well-established indication to filter placement. Prospective observational studies are needed to substantiate these findings.

## Figures and Tables

**Table 1:** Characteristics of the 168 patients who underwent IVCF placement.

Characteristics of Patients	Total Population (*n* = 168)
Age, years, median (interquartile range)	67 (55–76)
Male gender, *n* (%)	72 (42.8)
Previous history of VTE, *n* (%)	19 (11.3)
Atrial fibrillation, *n* (%)	11 (6.5)
Hypertension, *n* (%)	68 (40.5)
Diabetes mellitus, *n* (%)	27 (16.1)
Chronic kidney disease, *n* (%)	16 (9.5)
History of CAD, *n* (%)	8 (4.8)
History of CVD, *n* (%)	8 (4.8)
Chronic anemia, *n* (%)	10 (5.9)
Previous bleeding, *n* (%)	16 (9.5)
Previous or active malignancy, *n* (%)	99 (58.9)
Hematological disease, *n* (%)	11 (6.5)
Recent (<1 month) trauma, *n* (%)	34 (20.2)
Blood transfusion during hospitalization, *n* (%)	58 (34.5)
**Recent DVT (<4 weeks)**	
Proximal DVT, *n* (%)	144 (85.7)
Distal DVT, *n* (%)	24 (14.3)
**Indication to IVCF placement**	
Contraindication to anticoagulation, *n* (%)	116 (69.0)
Platelet count <25 × 10^9^/L, *n* (%)	20 (11.9)
Recent (<14 days) or active MB or CRNMB, *n* (%)	82 (48.8)
MB, *n* (%)	70 (41.7)
CRNMB, *n* (%)	12 (7.1)
Gastrointestinal, *n* (%)	24 (14.3)
Cerebral hemorrhage, *n* (%)	25 (14.9)
Metrorrhagia, *n* (%)	5 (3.0)
Hematuria, *n* (%)	3 (1.8)
Diseases/lesions with a high bleeding risk, *n* (%)	46 (27.4)
Urgent non-deferrable surgery, *n* (%)	101 (60.1)
With concomitant contraindication to anticoagulation, *n* (%) Without concomitant contraindication to anticoagulation, *n* (%)	49 (29.1) 52 (31.0)
**Blood exams before IVCF placement**	
Hemoglobin (g/dL), median (interquartile range) (normal range 13.0–17.0 g/dL)	10.5 (9.3–12.2)
Platelet count (10^9^/L), median (interquartile range) (normal range 150–450 × 10^9^/L)	245 (155–245)
Creatinine (mg/dL), median (interquartile range) (normal range 0.67–1.20 mg/dL)	0.8 (0.6–1.1)
**Patients on chronic antithrombotic therapy before IVCF placement**	
Antiplatelet, *n* (%)	15 (8.9)
Anticoagulant, *n* (%)	17 (10.1)
**Patients who received anticoagulant after IVCF placement**	
Full dose, *n* (%)	47 (28.0)
Under-therapeutic dose, *n* (%)	39 (23.2)
Prophylactic dose, *n* (%)	24 (14.3)
**Non-retrievable IVCF,** ***n*** **(%)**	71 (42.2)

Abbreviations: VTE: venous thromboembolism, CKD: chronic kidney disease, CAD: coronary artery disease, CVD: cerebrovascular disease, IVCF(s): inferior vena cava filters, DVT: deep vein thrombosis, CRNMB: clinically relevant non-major bleeding.

**Table 2:** Differences in continuous variables between patients who died and did not die during hospitalization.

Characteristics	Dead during Hospitalization (*n* = 24)		Discharged Alive (*n* = 144)		*p*-Value
	Median	IQR	Median	IQR	
Age, years	76	61–80	66	55–75	0.060
Hemoglobin, g/dL	9	8.1–11	11	9.5–12.3	**0.008**
Platelet count, 10^9^/L	181	103–291	248	163–351	0.056
Creatinine, mg/dL	1.0	1.0–1.4	1	0.6–1.0	0.970

**Table 3:** Differences in binary variables between patients who died and did not die during hospitalization.

Characteristics	Dead during Hospitalization (*n* = 24)	Discharged Alive (*n* = 144)	OR (95% CI)	*p*-Value
Male gender, *n* (%)	7 (29.2)	65 (45.1)	0.5 (0.2–1.28)	0.149
Previous history of VTE, *n* (%)	3 (12.5)	16 (11.1)	1.14 (0.31–4.26)	0.842
Hypertension, *n* (%)	13 (54.2)	55 (38.2)	1.91 (0.8–4.57)	0.144
Diabetes Mellitus, *n* (%)	3 (12.5)	24 (16.7)	0.71 (0.2–2.59)	0.608
Chronic kidney disease, *n* (%)	6 (25)	10 (6.9)	4.47 (1.45–13.76)	**0.009**
History of CAD, *n* (%)	3 (12.5)	5 (3.5)	3.97 (0.88–17.85)	0.072
History of CVD, *n* (%)	3 (12.5)	5 (3.5)	3.97 (0.88–17.85)	0.072
Chronic anemia, *n* (%)	1 (4.2)	9 (6.3)	0.65 (0.08–5.39)	0.692
Previous bleeding, *n* (%)	1 (4.2)	15 (10.4)	0.37 (0.05–2.97)	0.352
Previous or active malignancy, *n* (%)	14 (58.3)	85 (59)	0.97 (0.4–2.34)	0.949
Hematological disease, *n* (%)	2 (8.3)	9 (6.3)	1.36 (0.28–6.73)	0.703
Recent trauma, *n* (%)	8 (33.3)	26 (18.1)	2.27 (0.88–5.86)	0.091
Blood transfusion, *n* (%)	15 (62.5)	43 (29.9)	3.91 (1.59–9.63)	**0.003**
**Recent DVT (<4 weeks)**				
Proximal VTE	21 (87.5)	123 (85.4)	1.19 (0.32–4.36)	0.393
Distal VTE	3 (12.5)	21 (14.6)	0.83 (0.23–3.06)	0.393
**Indication to IVCF placement**				
Contraindication to anticoagulation	22 (91.7)	94 (65.3)	5.85 (1.32–25.9)	**0.020**
Platelet count <25 × 10^9^/L	5 (20.8)	15 (10.4)	2.26 (0.74–6.94)	0.153
Recent bleeding (<14 days)	17 (70.8)	65 (45.1)	2.95 (1.15–7.55)	**0.024**
Major	14 (58.3)	56 (38.9)	2.2 (0.91–5.29)	0.078
CRNMB	3 (12.5)	9 (6.3)	1.22 (0.25–5.94)	0.807
Gastrointestinal	7 (29.2)	17 (11.8)	3.08 (1.11–8.49)	**0.030**
Cerebral hemorrhage	3 (12.5)	22 (15.3)	0.79 (0.22–2.88)	0.724
Metrorrhagia	1 (4.2)	4 (2.8)	1.52 (0.16–14.23)	0.713
Hematuria	1 (4.2)	2 (1.4)	3.09 (0.27–35.44)	0.365
High bleeding risk diseases/lesions	8 (33.3)	38 (26.4)	1.39 (0.55–3.52)	0.481
Only urgent non deferrable surgery	2 (8.3)	50 (34.7)	0.17 (0.04–0.76)	0.02
**Antithrombotic therapy**				
Chronic	8 (33.3)	24 (16.7)	2.5 (0.96–6.5)	0.060
Anticoagulant after IVCF placement				
Full dose	4 (16.7)	43 (29.9)	0.47 (0.15–1.46)	0.191
Reduced dose 50–75%	3 (12.5)	36 (25)	0.43 (0.12–1.52)	0.190
Prophylactic dose	4 (16.7)	20 (13.9)	1.24 (0.38–4.01)	0.719
**Not retrievable IVCF**	12 (50)	59 (41)	1.44 (0.61–3.43)	0.409

Abbreviations: VTE: venous thromboembolism, CKD: chronic kidney disease, CAD: coronary artery disease, CVD: cerebrovascular disease, IVCF(s): inferior vena cava filters, DVT: deep vein thrombosis, CRNMB: clinically relevant non-major bleeding.

**Table 4:** Multivariate logistic regression analysis conducted using variables that were statistically significant in the univariate analysis in Table 2 and Table 3.

Characteristics	OR (95% CI)	*p*-Value
Chronic kidney disease	3.74 (1.13–12.32)	**0.030**
Contraindication to anticoagulation	4.26 (0.93–19.64)	0.063
Blood transfusion during hospitalization	2.97 (1.16–7.60)	**0.023**

## Data Availability

The raw data supporting the conclusions of this article could be made available by the authors.
